# Translating Metaphtonymy: Exploring Trainee Translators' Translation Approaches and Underlying Factors

**DOI:** 10.3389/fpsyg.2021.629527

**Published:** 2021-06-30

**Authors:** Shengxi Jin, Zhengjun Lin, Todd Oakley

**Affiliations:** ^1^The Preparatory School for Chinese Students to Japan, The Training Center of Ministry of Education for Studying Overseas, Northeast Normal University, Changchun, China; ^2^School of English and International Studies, Beijing Foreign Studies University, Beijing, China; ^3^Department of Cognitive Science, Case Western Reserve University, Cleveland, OH, United States

**Keywords:** metaphtonymy, metaphor, metonymy, cognitive translation, literary translation

## Abstract

Metaphtonymy is identified as a special rhetoric figure that specifies the interaction between metaphor and metonymy and which is pervasive in literary works. How and why do trainee translators translate metaphtonymy? Using task analysis, semi-structured discourse-based interviews, and a questionnaire survey among 30 master of translation and interpreting (MTI) trainee translators, this study investigates their translation approaches adopted when translating the metaphtonymies in Chinese extracted prose and explores the effects of their choices. It is found that they mainly employed three approaches: omission, modification, and retainment, with omission being the most, and retainment the least frequent. The main factors attributing to each approach range from the prominence degrees and cross-cultural adaptation abilities of the metaphtonymies, rhetorical awareness of translators, and transference competence to their translation knowledge sub-competence. This study suggests that trainee translators should be instructed to systematically construct rhetoric knowledge, and the teaching design should emphasize the competence of trainees of identifying rhetorical devices and their competence of shifting rhetoric between languages.

## Introduction

Since the 1980s, metonymy and metaphor have been widely accepted as different ways of thinking rather than as mere stylistic devices (Lakoff and Johnson, 1980; Panther and Radden, 1999; Dirven, 2003; Littlemore, 2019) and have received considerable attention within the studies across languages and cultures (Buchowski, 1996; Sakuragi and Fuller, 2003; Chen and Lai, 2012). As two modes of conceptualizing the world, they often interact with each other, prompting Goossens (1990) to offer the neologism: metaphtonymy. Predominant in literary works, metaphtonymies bear rhetorical forces that enhance poetic quality and aesthetic values while equally stimulating imaginations of the readers (Jin, 2019). Though figurative language, such as metaphor and metonymy, has been recognized as potentially challenging in translating literary works (Schäffner, 2004; Tymoczko, 2004; Tan, 2010), translation of metaphtonymy has received limited attention.

Handling metaphtonymies appropriately greatly affects the quality of the rendered texts of literary works and helps target-language readers gain a better insight into the culture of the source language. However, metaphtonymies are language-specific and elicit discrepancies in the identification of their patterns, the location of the source, and target domains in both the source and target languages (Goossens, 1990; Jin, 2019), thereby presenting translators with significant challenges. To gain a better insight into the translation of metaphtonymy, it is necessary to explore both how translators handle metaphtonymies and the reasons for the translating approaches adopted by them. Therefore, the present study is positioned within translation studies and focused on attempts of trainee translators to translate metaphtonymic language. Before proceeding to a detailed analysis, we wish to clarify some basic concepts and review the literature closely related to the study.

## Metaphor, Metonymy, Metaphtonymy, and Translation

The traditional approach viewed metaphor and metonymy as stylistic devices, largely the province of poets, politicians, and other public orators. Metaphor and metonymy are viewed as linguistic expressions, which are substituted for another expression in terms of “Similarity” or “Contiguity” (Lakoff and Johnson, 1980), respectively. While cognitive linguistic research has argued that language is structured by metaphorically and metonymically conceptual processes, which provides a new standard to judge whether a linguistic unit can be labeled as metaphor *via* conceptual mapping between two different conceptual domains: the target domain and the source domain (Lakoff and Turner, 1989; Littlemore, 2019) or metonymy *via* conceptual mapping between two different conceptual entities within a cognitive domain (Dirven, 2003; Littlemore, 2015). This extends the scope of linguistic metaphor and metonymy which exist in discourse.

Can a clear distinction be made between metaphor and metonymy? A linguistic expression cannot be identified as metaphor or metonymy in an absolute sense because the metaphoricity and metonymicity are decided by context, in other words, they are language-user-relative (Ruiz de Mendoza Ibáñez and Mairal Uson, 2007; Barnden, 2010). Taking literary works as an example, writers can use a metonymic expression to compare some other entity, which shares similarity with the vehicle, or a metaphorical expression to substitute some other entity that possesses a contiguous relationship with the vehicle. Thus, the connections between metaphor and metonymy are made more slippery (Barnden, 2010). The interaction between metaphor and metonymy is assigned to a cover term metaphtonymy by Goossens (1990). Metaphtonymy sits along a continuum at one end of which metaphors are highlighted and at the other, metonymies are prominent by which metaphtonymy is classified into two types: metaphor-metonymy and metonymy-metaphor (Jin, 2019). In the classical rhetorical tradition of Aristotle, Cicero, and Quintilian, metaphtonymy has a faint analog in *metalepsis*, as exemplified in “Pallid Death,” where the personification of death is highlighted by the synecdochic white-bloodless pallor of a dead person, devoid of circulation functions as a diagnostic feature of persona of death. Metaphtonymy is the preferred term, in this study, as it highlights the continual nature of the metaphor-metonymy axes not explicitly acknowledged in the classical tradition.

The phenomena of metaphor and metonymy have regularly been a concern to translation scholars and were explored in translating a variety of genres, including novels (Brdar and Brdar-Szabó, 2013), poetry (Lahiani, 2009; Jin, 2019), scientific texts (Merakchi and Rogers, 2013; Shuttleworth, 2017), political texts (Schäffner, 2004; Ghazala, 2012), and advertisements (Smith, 2006). The above research centers on the translation of metaphor and metonymy in terms of equivalence or non-equivalence in the target language for that of the source language, reflecting a prescriptive bias for the way to handle metaphor and metonymy. However, few translation studies investigate the interaction between metaphor and metonymy within a unified framework, which is the purpose of the present study.

Descriptive translation studies can undertake a more realistic approach and help reveal metaphtonymic regularities of inherence in interlinguistic transfer. Therefore, the study aims to explore the translation approaches of metaphtonymy and factors which contribute to choices of translators in the context of literary translation by employing task analysis and semi-structured discourse-based interviews, an analytical framework put forward by Peterlin and Moe (2016) in analyzing hedging translation and a questionnaire designed for the present study. Philip (2019:131) argues that studying the work of trainee translators offers a new perspective to metaphor translation because “the analysis of multiple translations of a given source text (ST) offers ample evidence of the strategies that are used in translation and allows researchers to incorporate aspects of language proficiency into their analysis.” Therefore, the present study addresses the translation of metaphtonymies in the context of translator training by focusing on the approaches of trainee translators to translating metaphtonymies in Chinese literary works into English. The study seeks to address two questions:

1. What are the translation approaches adopted by the trainee translators?
2. What affects the trainee translators' translating metaphtonymies?

## Research Design

### Participants

The participants involved in the study were 30 MTI students (26 females and 4 males), with ages ranging from 23 to 25 years (*M* = 24.23 and *SD* = 0.67), from the School of Foreign Languages, Northeast Normal University, each of whom was enrolled in the second year. All the participants were native speakers of Chinese and had learned English for at least 15 years. They all passed the Test for English majors–Band 8 organized annually by the National Foreign Languages Teaching Advisory Board under the Ministry of Education of the People's Republic of China (hereinafter abbreviated as PRC), which proves that they have acquired high-level competence in the English language and culture. Each participant demonstrated translation competence, obtaining a Level-2 certificate of the China Accreditation Test for Translators and Interpreters, a state-level vocational qualification examination entrusted by the Ministry of Human Resources and Social Security of the PRC. In addition, they have completed two courses in translation theory and four practical courses in translation strategy, literary translation, English and Chinese rhetoric and Chinese grammar, and rhetoric and writing. The participants provided their written informed consent forms prior to the translation task and were rewarded with course credits. All participants were informed that their translations were only for academic use.

### Data Collection

#### Translation Task

All the participants attended the translation workshop, an optional course designed and taught in the last term of the second year for students of MTI. They were from Class One opened by the first author. At the end of the term, they were given the task of translating the selected Chinese prose into English. The PDF version of the material (the ST) was sent to each participant *via* E-mail, and they were given 3 days to finish the translation task before the deadline. Their translations (the target text, hereinafter abbreviated as the TT) were submitted in word documents *via* E-mail.

The translations of participants were to be used to assess their achievements in the course. Each participant had access to the internet and the library for information related to the prose and the writer. The participants could refer back to translation dictionaries when needed. It was assumed that the participants would be familiar with the genre of prose, as literary works including prose had been used as STs in their translation classes.

#### Translation Materials

Consulting the criteria for evaluating interpreting materials proposed by Zheng and Xiang (2014), we made three criteria for selecting the translation materials. First, the length of the ST should be within that of their regular translation assignments. The ST was abridged prose from *Cultural Sojourn* written by Yu Qiuyu, one of the most influential contemporary Chinese writers. As important prose of his volume *Cultural Sojourn* published in 1992, the full text of the prose was longer than their previous translation assignments, thus, the ST was abridged from Section One and the first five paragraphs of Section Two, consisting of 1,403 words. Second, the ST should be the one that has never been translated into English. The selected prose meets this criterion, and no translations would be used as a reference by the participants. The last criterion is related to the number of metaphtonymies in the ST. Ten metaphtonymies were identified based on the below-mentioned identification procedure.

#### Metaphtonymy Identification Procedure

As for the identification procedures, we modified MIPVU established by Steen et al. (2010) and Five-step Identification by Jin (2019) for, respectively, identifying metaphor and metonymy. Finally, we adhered to the above-mentioned definition of metaphtonymy and decided metaphtonymic expressions. The identification procedures are as follows: (1) read the whole text and get a thorough understanding of the ST, (2) locate the potential-source-domain lexical units by examining the text word-for-word, (3) detect the metaphoricity or metonymic attribute of the word or expression by comparing the contextual meaning and the basic contemporary meaning of the unit; if the basic meaning is connected with the contextual meaning *via* similarity, the linguistic unit is labeled as metaphor; if *via* contiguity, it is labeled as metonymy, (4) determine if the linguistic metaphor conveys metonymic meaning *via* conceptual mapping, or if the linguistic metonymy embodies metaphorical meaning *via* conceptual mapping. If it is one of the cases in (4), label the linguistic unit as a metaphtonymy. The third and fourth steps were also used to examine if the translations of metaphtonymies identified in the ST were metaphors, metonymies, or metaphtonymies. When deciding the basic meaning and contextual meaning of the linguistic unit, we referred to *Xin Hua Ci Dian*, a Chinese authoritative dictionary, and some journals analyzing the prose. With few exceptions, all the explanations of the metaphtonymic expressions rely on that source, and the exceptions will be identified as they appear. As per the procedure, 10 metaphtonymic lexical entries presented in Table 1 and explained in Appendix 3 were identified in the ST. The 30 TTs (TT_1_-TT_30_) were analyzed by the authors in terms of the way that metaphtonymies in the ST were translated by each participant, thus, translation approaches were categorized.

**Table 1:** Percentage distribution of translation approaches to each metaphtonymy.

**Metaphtonymic expressions**	**% of TTs adopting retainment approach**	**% of TTs adopting modification approach**	**% of TTs adopting omission approach**
官屠宰辅 *guan tu zai fu*	6.7	13.3	80.0
侠骨赤胆 *xia gu chi dan*	10.0	33.3	56.7
蝇营狗苟 *ying ying gou gou*	13.3	56.7	30.0
脂腻粉渍 *zhi ni fen zi*	6.7	23.3	70.0
钢笔文化 *gang bi wen hua*	83.3	0.0	16.7
五四斗士 *wu si dou shi*	66.6	6.7	26.7
得心应手 *de xin ying shou*	6.7	20.0	73.3
艺术人格 *yi shu ren ge*	73.4	13.3	13.3
谈吐行止 *tan tu xing zhi*	0.0	0.0	100.0
墨香 *mo xiang*	53.3	36.7	10.0

#### Questionnaire and Interview

A questionnaire was given to all the participants before they handed in their translations, which was returned to the researchers with their translations. The questionnaire consists of 14 questions focusing on their knowledge of metaphor, metonymy, and metaphtonymy (3 questions), their identification of metaphor, metonymy, and metaphtonymy in the translation task (3 questions), their attitudes toward handling the metaphtonymies in translation (4 questions), and the challenges they met in translating those figurative linguistic units (4 questions). They could refer back to their translations when they filled in the questionnaire.

During the next day, 10 participants (one-third of the group) were randomly selected to attend a retrospective discourse-based semi-structured interview. In the interview, each participant (named as P_1_-P_10_) was given back their translations and the ST for reference. Metaphtonymies and their translations (if translated by the participant) have been highlighted with a red line by the authors in the ST and their translations. Subsequently, each of them was asked to explain what metaphor and metonymy are, and then the interviewer elaborated what metaphtonymy meant to them. Finally, the participants were encouraged to reflect on their translating processes, especially, the processes of translating the highlighted linguistic units, and above all the reasons why those metaphtonymies were handled that way. The interviews were organized in Chinese and finally were recorded and transcribed. In this study, each question of the interviewer and the response of the interviewee has been translated into English by the authors.

## The Approaches Adopted by the Trainee Translators

The metaphtonymic expressions in the ST are identified *via* mapping between domains and the interaction between metaphor and metonymy. The manner of handling the mapping of the trainees and the interaction embodied in the metaphtonymic expressions in the ST constitutes the criteria to identify trainee methodology. After an analysis of all the TT, three approaches were summarized and classified as retainment, modification, and omission. Table 1 lists the metaphtonymic expressions and the percentage of each approach adopted in the TT. In general, the three approaches are the same as the ones proposed by Schäffner (2004) to handle metaphors and by Brdar and Brdar-Szabó (2013) to translate metonymies but differ in specific procedures.

Retainment is defined as a literal translation of the metaphtonymic expressions in the ST. Specifically, this approach emphasizes sustaining the mapping and interaction between metaphor and metonymy and transfers them to the TT. This approach is illustrated in examples 1–3.

ST1: 五四斗士 们自己也使用毛笔，但他们是用毛笔在呼唤着钢笔文化 。

*Wu si dou shi men zi ji ye shi yong mao bi, dan shi ta men shi yong mao bi zai hu huan zhe gang bi wen hua*.

TT: Example 1. The May Fourth warriors themselves also used Chinese brush pens, but just regarded them as tools to call the Pen Culture.

Example 2. The May Fourth fighters used brushes themselves, but they used them to call forth the Pen Culture.

Example 3. The soldiers of May Fourth also wrote in brush, but they called for pen culture with their brushes.

In the above ST, “五四斗士” (*wu si dou shi*), is a metaphtonymic expression, in which “斗士” (its basic meaning is fighters or warriors), metonymically stands for the Chinese patriotic demonstration initiated on May 4, 1919, and “五四” (its basic meaning is May Fourth) metaphorically refers to the demonstration camp mainly constituted by college students, intellectuals and citizens, etc. “斗士” and “五四” interact conceptually with each other and combine into a phrase. The three translations keep the conceptual interaction relation by literally translating “五四” into May Fourth and “斗士” into fighters, soldiers, or warriors. Table 1 shows that the retained-approach translation of “五四斗士” amounts to 66.6%, which ranks after that of “钢笔文化” and “艺术人格.”

Modification occurs when the metaphtonymic expression was translated sustaining the metaphorical element in the ST or using a novel metaphtonymic expression to substitute the metaphtonymically conceptual relations in the ST.

ST2: 不管 他 们是……还是 蝇营狗苟 ，… 这副笔墨总是有的。

*Bu guan ta men shi… hai shi ying ying gou gou,…zhe fu bi mo zong shi you de*.

TT: Example 4. Whether they were …or flies-like cowards, they possessed a writing brush and ink anyway.

Example 5. Whether they were…or piggy Shylock,…they always took this pair of writing brush and ink.

In ST2, the metaphtonymic attribute of “蝇营狗苟” was quoted by Yu Qiuyu from one of the poems written by Han Yu, a famous poet in the Tang Dynasty. The Chinese words “蝇” (its basic meaning is fly) and “狗” (its basic meaning is dog) comprise the source domain and map onto the target domain of greedy and duplicitous people, and Han Yu metonymically used the phrase to name his colleagues who served the royal families. Example 4 kept one of the metaphors in the ST using “flies-like cowards,” while Example 5 creatively replaced the metaphtonymic relations in the ST adopting “piggy Shylock” in which a new interaction occurs between a new pair of metaphor and metonymy. The word “piggy” metaphorically prompts the source domain “pig” in which some bad features of pigs, such as laziness and greed, are mapped onto the target domain of avaricious people. Shylock, a classical figure in Shakespeare's *The Merchant of Venice*, is widely accepted as a metonymy standing for cunning and greedy men. The phrase “piggy Shylock” embodying metaphtonymic relations was used by the trainee translator as the equivalent of metaphtonymy of the ST.

Omission occurs through ellipsis, where the translation of the ST offers no metaphorical and metonymic equivalents that are employed in the TT, thus leading to the deletion of the interaction between metaphor and metonymy in the TT.

ST3: 文人们的衣衫步履、 谈吐行止 、 居室布置、交际往来 ，都与书法构成和谐 。

*Wen ren men de yi shan bu lv, tan tu xing zhi, ju shi bu zhi, jiao ji wang lai, dou yu shu fa gou cheng he xie*.

TT: Example 6. The literati's dressing codes and behavioral manners, living conditions and communication habits were all in harmony with calligraphy.

Example 7. The literati's living styles and social interactions were all in harmony with calligraphy.

In ST3, the metaphoricity of “谈吐行止” resides in the compound “谈吐,” in which the word “吐” means throwing up something out of the mouth of an individual. The basic meaning of “谈” is talking, and combined with “吐,” “谈吐” means making utterances. The abstract content of these utterances is metaphorically contrasted with some specific things thrown out of the mouth. In “行止,” “止,” an interchangeable word with “趾,” whose meaning is toes, first word stands for foot, then refers to walking manners and is finally combined with “行” to metonymically mean behavior patterns, thereby forming a metonymic chain. “谈吐” and “行止” form a phrase emphasizing the interactive relationship between metaphor and metonymy. Example 6 employs “behavioral manner,” the metonymic meaning of “谈吐行止.” While Example 7 uses “living styles,” an umbrella term, to refer to “dressing code,” “behavior manner,” and “room layout,” which are denoted by “衣衫步履,” “谈 吐行止,” “居室布置,” respectively. The two examples adopt the omission approach to translate the metaphtonymic phrase “谈吐行止” in the TT.

Table 1 presents different distribution percentages of translation approaches employed by the trainee translators to handle each metaphtonymy from the ST among the 30 TTs. In Table 2, variables, the metaphtonymic expressions, and translation approaches are specified. The former table is listed as to the sequence in which they appear in the ST, and the latter is summarized as approaches of retainment, modification, and omission. Table 1 shows significant differences in the percentage of each approach in relation to each metaphtonymic expression, the highest rate being 100% of omission to “谈吐行止,” while the lowest rate being 0% of modification to “毛笔文化,” “谈吐行止,” and “毛笔文化,” and retainment to “谈吐行止.”

**Table 2:** Percentage of each approach in each target text (TT).

**TT**	**% Retainment approach**	**% Modification approach**	**% Omission approach**
TT_1_	30.0	40.0	30.0
TT_2_	0.0	30.0	70.0
TT_3_	10.0	40.0	50.0
TT_4_	20.0	20.0	60.0
TT_5_	10.0	40.0	50.0
TT_6_	10.0	30.0	60.0
TT_7_	20.0	40.0	40.0
TT_8_	20.0	30.0	50.0
TT_9_	10.0	30.0	60.0
TT_10_	20.0	10.0	70.0
TT_11_	10.0	50.0	40.0
TT_12_	10.0	40.0	50.0
TT_13_	20.0	20.0	60.0
TT_14_	20.0	40.0	40.0
TT_15_	10.0	30.0	60.0
TT_16_	20.0	30.0	50.0
TT_17_	10.0	30.0	60.0
TT_18_	40.0	20.0	40.0
TT_19_	20.0	10.0	70.0
TT_20_	20.0	40.0	40.0
TT_21_	20.0	30.0	50.0
TT_22_	20.0	20.0	60.0
TT_23_	0.0	40.0	60.0
TT_24_	20.0	50.0	30.0
TT_25_	10.0	40.0	50.0
TT_26_	20.0	20.0	60.0
TT_27_	30.0	10.0	60.0
TT_28_	10.0	30.0	60.0
TT_29_	20.0	30.0	50.0
TT_30_	30.0	20.0	50.0
**Average**	**17.0**	**30.3**	**52.7**

Besides the different rates of translation approaches to each metaphtonymy, every trainee translator shows considerable variations in the three approaches employed in their TTs, which are listed in Table 2. Precisely, the percentage of all the 10 metaphtonymic expressions either retained, modified, or deleted in each TT is distinct.

Table 2 shows omission, as the most frequent approach, followed by modification and retainment. In TT_2_ and TT_23_, retainment was not adopted.

In all the 30 TTs, the omission approach was more frequently employed to translate the metaphtonymic expressions including “官屠宰辅,” “侠骨赤胆,” “脂腻粉渍,” “得心应手,” and “谈吐行止.” These four-character structures constitute idioms originating from some Chinese historic allusions, which meant challenges to the thirty translators because they are culture-specific, and find no equivalents in target language culture. However, analysis of the translations reveals that most translators had a good command of the contextual meanings of these metaphtonymic expressions but chose to omit their metaphtonymic provenance.

The modification approach accounts for nearly one-third of the 30 TTs. It is assumed that most translators attempted to sustain the rhetoric force in the ST using the equivalent metaphor or metonymy or modified metaphtonymic expressions in the TT.

Retainment was used by 28 trainee translators to render “钢笔文化,” except the two of TT_2_ and TT_23_ discussed. These two examples presented the metonymic meaning of “钢笔文化” and translated them explicitly as “foreign advanced culture” and “the great culture of western countries.”

ST4: 五四斗士们自己也使用毛笔，但他们是用毛笔在呼唤着钢笔文化 。

*Wu si dou shi men zi ji ye shi yong mao bi, dan ta men shi yong mao bi zai hu huan zhe gang bi wen hua*.

TT: Example 8. The fighters of May Fourth Movement used brushes, but they were using them to call upon foreign advanced culture.

Example 9. The warriors of the Movement also used brushes, however, they called for the great culture of western countries.

## Three Types of Comments Generalized From the Questionnaires and Interviews

The questionnaires and interviews centered on the translation process of metaphtonymic expressions of the participants, with the intention to probe how they identified metaphtonymies of the ST, reveal their attitudes toward translating these rhetorical figures of the ST and understands the difficulties they encountered when translating these figurative languages.

### Comments on the Identification of Metaphtonymies of the ST

In the questionnaire, questions 1–6 were related to their understandings of metaphor, metonymy, metaphtonymy and whether they could identify them in the ST. Nearly 93% of the participants reported that they had sufficient knowledge of metaphor and metonymy, the rest reported that they had a basic knowledge of metaphor and metonymy, while 80% of the answers of the participants revealed that they knew metaphtonymy but seldom encountered, analyzed, or relied on them in practice. Almost 70% of the participants admitted that they knew what metaphtonymy and its working mechanism were because they translated some cognitive linguistics papers in which they saw this term and referred back to common dictionaries and journals. The rest explained that they found some lexical units entailed both metaphorical meaning and metonymic meaning after referring to dictionaries and analyzing the context in which these lexical units emerged, but they did not know the term used to name these phenomena.

All the participants reported that the ST was well-written in which metaphors and metonymies were widely used. When asked to list some specific examples, they could give at least five for each group. However, as to metaphtonymies, most of them could list “官屠宰辅,” “侠骨赤胆,” “蝇营狗苟,” “脂腻粉渍,” or ‘五四斗士,” while a few participants categorized “蝇营狗苟” into metaphor, but “脂腻粉渍” and “官屠宰辅” into metonymies. Three of the participants in the interview explained that the metaphoricity of “蝇营狗苟” was highly prominent, for instance:

Interviewer: ‘为什么你认为‘ 蝇营狗苟 '是隐喻?” [Why did you identify “蝇营狗苟” as a metaphor?]

P_4_: “很明显, 这是个隐喻。在文中，作者把部分传统文人比喻成 ‘苍蝇 '或 ‘狗'。查 了相关资料 后 ，才 了解到这 短语 是比喻不择手段追求名利的人. [Obviously, it is a metaphor. In the prose, the author compared some of the Chinese literati with flies or dogs. After referring to some materials, I knew its extended metaphorical meaning is to name those who earn names or make fortunes by unfair means or foul.]

All the interviewees reported that they first searched for in their mental lexicon the meaning of the 10 metaphtonymies highlighted in their TTs by the teacher, and then judged the meaning based on linguistic context. If they were uncertain, they continued to look it up into dictionaries or the internet to find out its basic and extended meaning and then reanalyzed the sentence in the ST. After the meaning of the lexical unit was decided, they would search for its equivalent in the TT. Taking the response of Participant 9 as an example:

Interviewer: “能 请 你 回忆下隐喻转喻互动表达“五四斗士”的翻译过程 吗? [Could you please retrospect your translating process of the metaphtonymic expression “五四斗士”?]

P_9_: “读到这个句子, 就想到 ‘五四 '是指 ‘五四运动 ', 斗士是比喻那些 运动 领导 者和 参与者。虽然五四运动的历史 早 就学过, 但 译前 还是 上网 查阅了相关介绍 。 在英语里斗士对应的词有 soldier, warrior 等, 查阅 字典 后, 我选择了 soldier, 因为 它的语义色彩更积极。五四运动上文已经提过, 所以译为 The soldiers of May Fourth 不会让英语读者困惑。” [It occurred to me that May 4 refers to May Fourth Movement upon reading the sentence. The word “斗士” is used metaphorically to refer to the leaders and participants of the Movement. I learned the Movement history, but I reviewed it online before translation. In English, soldier and warrior are equivalents of “斗士”. But after referring to dictionaries, I chose soldier because its meaning is more positive. May Fourth Movement was mentioned at the beginning of the paragraph. Therefore, my translation “May Fourth” would not confuse target language readers.]

### Comments on the Importance of Translating Metaphtonymies of the ST

As to the rhetorical devices of the ST, the questionnaires show that nearly 33.3% of the participants thought it was extremely important to sustain or modify the figurative languages in the TT, those who thought it was important or unimportant accounted for, respectively, 53.3% or 13.4%. Overall, most participants attached importance to the metaphtonymies in the TT in terms of their equivalents of the ST. Their responses show that they were more concerned with the equivalence of stylistic features between the ST and the TT.

They thought the ST was prose, characterized by highly poetic language, for which it was the duty of translator to preserve. The response of participant 1 is instructive in this respect:

Interviewer: “你认为在译文中保留原文中的转喻、隐喻等修辞重要吗？为什么？” [Do you think it is important to sustain the rhetorical devices of the ST, say, metaphor and metonymy? And why?]

P_1_: “我认为保留原文中的修辞很重要。 我也是这么做的。 例如， 在英语里找 不到 “蝇营狗苟 ”的对应项，我就用了 “piggy Shylock” 这个转喻来代替, 这也算创造性翻译吧 。 原文是散文，语言美，修辞多，意境 还很 深。如果翻译的时候，不保留这些特征，只用 简单 语言，译文就不美了，不能表达余秋雨的写作意图了。 ” [I think it is a very important standard and I followed that in my translation. For example, I creatively used “piggy Shylock” to translate ‘蝇营狗苟' because I could not find its equivalent in the TT. The ST is prose. It consists of beautiful languages and rich rhetorical devices, and it also reveals a deep poetic world. If these features were lost in the TT, my translation would lose its beauty and could not realize the writing purpose of ST author.]

### Comments on the Challenges of Translating Metaphtonymies of the ST

About 86.6% of the participants thought it was necessary to translate figurative languages into their equivalents in the TT. However, Table 2 shows that most of them relied on the omission to translate the metaphtonymic expressions of the ST, and a minority of them retained the ST metaphtonymies. Why did their practice contradict their stated principle? The reasons seem to reside in the challenges they met during the translation process. First, nearly 73.3% of the participants in the questionnaires, in contrast with 60% in the interviews, reported that although they could identify some ST figurative expressions, they were not able to find their appropriate equivalents in English because those expressions, say, “官屠宰辅 ” and “蝇营狗苟,” are Chinese culture-specific. They had to paraphrase some of the figurative expressions and explicitly presented their metaphorical or metonymic meaning in the TT. Moreover, only 23% of participants in their questionnaires admitted that they did not identify some of the expressions as metaphors, metonymies, or metaphtonymies, but assumed them to be plain expressions, or only viewed some metaphtonymies as metaphors, among which “五四斗士” was the typical example mentioned by the participants. However, in the interview, the retrospective processes of three interviewees reveal that their understanding of “五四斗士” consisted of metonymic thinking and metaphorical thinking. Specifically, they knew the date of “五四” referred to the May Fourth Movement but were not aware that it was a metonymy, so they assumed “五四斗士” to be a metaphor because “斗士” was an obvious source metaphorically used to refer to the leaders and participants. Finally, the differences between Chinese and English language structures brought challenges to them when they translated those figurative languages, which were reported by 56.7% of participants in their questionnaires and 60% of interviewees in the interviews. For instance:

Interviewer: 源语和目的语存在差别, 这对你翻译包括隐喻转喻互动现象在内的修辞 产生影响了吗？ [Were there any influences from the differences between the source language and target language on your handling of the figurative languages including metaphtonymy?]

P_7_: 是啊, 有影响。汉语四字格结构, 对仗优美, 比如说这篇散文中的 “得心应手” 翻译的时候如果把 “心 ”和 “手 ”都翻译出来, 就会使英语句子冗长, 不符合英语的习惯。所以, 要么保留一个, 要么意译. [Definitely yes. The Chinese language has many four-character phrases whose internal structures are beautifully antithetical. When translating them, say, “得心应手” in this prose, I had to retain “心” or “手” or translate its extended meaning because retaining both of them in the TT would make the English translation redundant and that was inconsistent with English usage.]

## The Underlying Factors Affecting the Translators Translating Metaphtonymies

The analysis of the TT translated by the trainee translators revealed that three main approaches were employed, namely, retainment, modification, and omission, although there were some variations among the individual participants to some extent. Combined with the analysis of the translations of participants, the results of the questionnaires and interviews show that there were at least three underlying factors affecting how the participants handled these metaphtonymic expressions in the TT.

### Restrictions From the Metaphtonymic Expressions

The first factor contributing to the three translation approaches was metaphtonymic expressions, specifically, their prominence degrees in the ST and cross-cultural adaptation abilities in the TT. Figures of rhetoric occur when an expression deviates from expectation of the receiver, but the expression is still interpreted as an appropriate one (McQuarrie and Mick, 1996). First, most of the metaphtonymic expressions in the translation task are idioms embodying metaphorical and metonymic meanings. They are Chinese ancient culture-specific and thus highlighted in the linguistic context established by contemporary Chinese lexical entities and grammars. The more cultural connotations they entail, the higher prominence degrees they show. Naturally, the translators paid more attention to those metaphtonymies, which required greater cognitive effort. Moreover, cross-cultural adaptation abilities of the metaphtonymies also exert influence on the choices of translators. The cross-cultural adaptation ability of a lexical entry refers to whether it, in the target language system, has an equivalent which can be used directly by translators in the TT. The one whose equivalent is available in the TT has stronger cross-cultural adaptation ability, which means it has a lower degree of difficulty for translators. Retainment was most frequently employed to render those metaphonic expressions, which possess high prominence degrees and stronger cross-cultural adaptation abilities. That seems to explain why “五四斗士” was rendered retaining its metaphtonymic meaning but “官屠宰辅” translated deleting or modifying its metaphtonymic meaning.

### Restrictions From Rhetoric Awareness and Transference Competence

The second factor was metaphtonymy awareness of the trainee translators and their transference competence of bilingual rhetoric. The competence of translators in recognizing the rhetorical devices of the ST when translating figurative language requires them to adopt appropriate strategies (Smith, 2006), which suggests that translators should be aware of the type of rhetorical figures and their working mechanisms. What the participants responded to the questions revealed that most of them could consciously locate and identify the rhetoric devices including metaphtonymies during the compression process of the ST, which showed that they had high rhetorical awareness, specifically, the awareness of identifying and processing the rhetorical expressions. Their rhetorical awareness in translation seemed to be creditable for at least three aspects. The first, and most important, aspect was that they acquired basic knowledge of translating rhetoric because they had attended relevant courses. Another prominent aspect was the stylistic features of the translation materials. In their previous translation courses and practice, all the participants have been instructed to pay more attention to the stylistic features of the ST before translation so that they could have a better understanding of translation materials and decide what translation strategies would be adopted. The translation material in the present study was an extracted prose, which could arouse the interest of the participants. Last but not least, most of the metaphtonymies of the ST are Chinese culture-specific, and some are idioms. The participants could have access to the location and identification of those rhetorical devices during the comprehension stage. The identification of metaphtonymies of the ST by the translators constituted the preconditions by which they could employ felicitous strategies to handle these figurative languages.

The bilingual sub-competence, a very basic componential element of translation competence, is made up of pragmatic, socio-linguistic, textual, and lexical-grammatical knowledge in each language (PACTE GROUP, 2005; Göpferich, 2013). The transference competence of translators of source rhetoric falls within this category. Not all the participants had strong transference competence of rhetorical devices. Four of the interviewed participants, accounting for 40% of the total, reported that they could not find the equivalents because they lacked socio-linguistic or lexical-grammatical knowledge related to the ST metaphtonymies in the target language, leading to their modification or deletion of the ST metaphtonymic meanings. Therefore, their translations of metaphtonymies were, in general, limited to the omission approach, which worsened, to a larger extent, their strategic sub-competence regarded by the (PACTE GROUP, 2005:610) as “the most important and responsible for solving problems and the efficiency of the process.”

### Restrictions From Translation Knowledge Sub-competence of Translators

The last factor seemed to be the translation knowledge sub-competence of participants, namely, the knowledge of the principles of translators that guide their translation processes (PACTE GROUP, 2005). During the first 2 years, all the participants had acquired certain translation knowledge through attending translation courses, and large translation practices including a 3-month internship. They knew how to carry out a translation project and what translation principles are to be followed with respect to different genres and occasions. The translation knowledge they had, say, one of duties of translators to chase the rhetoric equivalence between the ST and TT in literary translation, dictates they are to retain the metaphtonymic meaning in the translation material. However, modification and omission were the dominant methods used under rhetorical equivalence.

## Conclusions

This study examined the performance of Chinese students of MTI in translating metaphtonymic devices in an extracted Chinese prose, based on the analysis of the TT, combined with questionnaires and interviews after translation. It was found that the participants most frequently deleted the interactive relations between metaphor and metonymy of the ST by explicitly presenting in the TT the extending meaning of the metaphtonymic expressions. Moreover, the participants modified the ST metaphtonymies as metaphors or metonymies in the TT or creatively employed a new English metaphtonymy. However, not all the participants attempted to retain the ST metaphtonymies in the TT. In other words, retainment was employed less frequently, a subtle violation of the rhetorical equivalence principle.

The analysis of the questionnaires and interviews shed light on the factors contributing to the three approaches employed by the participants to translate metaphtonymies. It seems that the three approaches were influenced by the prominent cultural features of the ST metaphtonymies and whether they had lexical equivalents in the target language system. In addition, the rhetoric awareness and rhetoric shifting intercultural competence of translators comprised the underlying reasons for the choices of translators in handling metaphtonymies. The principles and standards to which the translators adhered also exerted influence on the way they dealt with the ST metaphtonymies. It can be concluded that metaphorical and metonymic thinking of translators emerges when they comprehend and render the metaphtonymies.

The findings raise several implications for training translators. In training practice mainly related to rhetoric and translation, trainees should be instructed to systematically construct knowledge of the rhetorical structures, including analyzing metaphor, metonymy, and the interactions between them. In addition, teaching design should center on competence of trainees in identifying multiple rhetorical devices, and their competence in shifting rhetoric between languages. Instruction of trainees should emphasize the features of different genres of texts and the importance of mastering appropriate translating principles and strategies. The findings also provide potentially important issues for future studies. The translation material was narrowed down to Chinese prose, one type of literary work, but metaphtonymy is not the sole province of literature, it exists in all types of prose and is distributed over many different genres and occasions. Additional research should focus on scientific and political texts, to enhance the reliability and validity of the present findings. Moreover, the sample of trainee translators in the present study was in relatively small quantity and limited to Chinese students of MTI. Therefore, it is suggested that the follow-up research should consider comparative studies of novice and expert translators, with the hope of teasing out other factors contributing to the translation of metaphtonymies. Finally, methods extending beyond *ex-post-facto* task analysis and semi-structured interviews should be developed, such as think-aloud protocols and also eye-tracking protocols for capturing attention fixation points in the ST.

## Data Availability Statement

The original contributions presented in the study are included in the article/Supplementary Material, further inquiries can be directed to the corresponding author/s.

## Ethics Statement

Ethical review and approval was not required for the study on human participants in accordance with the local legislation and institutional requirements. Written informed consent for participation was not required for this study in accordance with the national legislation and the institutional requirements.

## Author Contributions

SJ designed the study and the manuscript, analyzed the data, and wrote all sections. ZL collected, annotated, and analyzed the data. TO reviewed and edited the manuscript. All authors contributed to the conception of the study and analyzed and discussed the data.

## Conflict of Interest

The authors declare that the research was conducted in the absence of any commercial or financial relationships that could be construed as a potential conflict of interest.
