# Supplementary material for: Translating Metaphtonymy: Exploring Trainee Translators' Translation Approaches and Underlying Factors
Source: Front Psychol. 2021 Jun 30;12:629527. doi: 10.3389/fpsyg.2021.629527 (PMC8278523; doi:10.3389/fpsyg.2021.629527)
Supplement: Supplementary file 1 [file Data_Sheet_1.pdf]

Appendix 1 Questionnaire

This questionnaire, designed for the translation task and used only for research, is anonymous and ungraded. Thanks for your participation.

Part one Basic information

Age ______ Gender ______ TEM ______ CATTI Level ______

Part two Questions

1. I have sufficient knowledge of metaphor. ______
   A. strongly disagree B. disagree C. not sure D. agree E. strongly agree
2. I have sufficient knowledge of metonymy. ______
   A. strongly disagree B. disagree C. not sure D. agree E. strongly agree
3. I have sufficient knowledge of metaphtonymy. ______
   A. strongly disagree B. disagree C. not sure D. agree E. strongly agree
4. It is important to identify the figurative language including metaphtonymies in the source text. ______
   A. strongly disagree B. disagree C. not sure D. agree E. strongly agree
5. It is important to retain figurative language including metaphtonymies in the target text. ______
   A. strongly disagree B. disagree C. not sure D. agree E. strongly agree
6. Appropriate handling of figurative language influences the quality of the target text. ______
   A. strongly disagree B. disagree C. not sure D. agree E. strongly agree
7. It is very challenging to handle figurative language in the translation task. ______
   A. strongly disagree B. disagree C. not sure D. agree E. strongly agree
8. I can competently identify the metaphors and metonymies in the translation task. ______
   A. strongly disagree B. disagree C. not sure D. agree E. strongly agree
9. I have strong competence of identifying the metaphtonymies in the translation task. ______
   A. strongly disagree B. disagree C. not sure D. agree E. strongly agree
10. I have sufficient language shifting capabilities to accommodate the metaphors and metonymies in the translation task. ______
    A. strongly disagree B. disagree C. not sure D. agree E. strongly agree
11. I have sufficient language shifting capabilities to accommodate the metaphtonymies in the translation task. ______
    A. strongly disagree B. disagree C. not sure D. agree E. strongly agree
12. Please list all the metaphors, metonymies, metaphtonymies you have identified in the translation task.
13. What are the specific challenges you face when translating the metaphors and metonymies in the translation task?
14. What are the specific challenges you face when translating the metaphtonymies in the translation task?

Appendix2 Interview questions

The introductory and closing statements are omitted. Nine of the questions are listed below due to length limitation.

1. Are you familiar with metaphor, metonymy and metaphtonymy?
2. Please comment on the figurative language in the translation material.
3. Did you identify all the metaphors, metonymies and metaphtonymies in the translation material?

4. What did you consult to help you understand the metaphors, metonymies and metaphtonymies in the translation materials?
5. Explain the process of handling the metaphtonymies (choose one of the ten highlighted with red line by the author in their source texts and translations) during the translation.
6. What methods did you use to translate the metaphtonymies in the translation task?
7. Why did you render the metaphtonymies in the translation task that way?
8. What are the contributing factors that influenced your translation of the metaphtonymies?
9. What translation principle(s) did you follow when translating figure language?

Appendix 3 Metaphtonymic expressions

<table>
<tr><td rowspan="4">1</td><td>官</td><td>屠</td><td>宰</td><td>辅</td></tr>
<tr><td>guan</td><td>tu</td><td>zai</td><td>fu</td></tr>
<tr><td>official</td><td>butcher</td><td>chancellor</td><td>assist</td></tr>
<tr><td colspan="4">‘very high-ranking officials’</td></tr>
<tr><td rowspan="4">2</td><td>侠</td><td>骨</td><td>赤</td><td>胆</td></tr>
<tr><td>xia</td><td>gu</td><td>chi</td><td>dan</td></tr>
<tr><td>knight</td><td>bone</td><td>red</td><td>gallbladder</td></tr>
<tr><td colspan="4">‘righteous and bold’</td></tr>
<tr><td rowspan="4">3</td><td>蝇</td><td>营</td><td>狗</td><td>苟</td></tr>
<tr><td>ying</td><td>ying</td><td>gou</td><td>gou</td></tr>
<tr><td>fly</td><td>swarm</td><td>dog</td><td>perfunctory</td></tr>
<tr><td colspan="4">‘swarm as flies do for good or hang round as dogs do for food’</td></tr>
<tr><td rowspan="4">4</td><td>脂</td><td>腻</td><td>粉</td><td>渍</td></tr>
<tr><td>zhi</td><td>ni</td><td>fen</td><td>zi</td></tr>
<tr><td>rouge</td><td>greasy</td><td>powder</td><td>stain</td></tr>
<tr><td colspan="4">‘those female literati’</td></tr>
<tr><td rowspan="4">5</td><td>钢</td><td>笔</td><td>文</td><td>化</td></tr>
<tr><td>gang</td><td>bi</td><td>wen</td><td>hua</td></tr>
<tr><td colspan="2">pen</td><td colspan="2">culture</td></tr>
<tr><td colspan="4">‘the culture represented by the pen’</td></tr>
<tr><td rowspan="4">6</td><td>五</td><td>四</td><td>斗</td><td>士</td></tr>
<tr><td>wu</td><td>si</td><td>dou</td><td>shi</td></tr>
<tr><td>May</td><td>four</td><td>fight</td><td>man</td></tr>
<tr><td colspan="4">‘those who initiated and attended the May 4 Movement’</td></tr>
<tr><td rowspan="4">7</td><td>得</td><td>心</td><td>应</td><td>手</td></tr>
<tr><td>de</td><td>xin</td><td>ying</td><td>shou</td></tr>
<tr><td>get</td><td>heart</td><td>respond</td><td>hand</td></tr>
<tr><td colspan="4">‘what the heart wishes, one's hands accomplish’</td></tr>
<tr><td rowspan="4">8</td><td>艺</td><td>术</td><td>人</td><td>格</td></tr>
<tr><td>yi</td><td>shu</td><td>ren</td><td>ge</td></tr>
<tr><td>skill</td><td>approach</td><td>man</td><td>quality</td></tr>
<tr><td colspan="4">‘personality of art’</td></tr>
</table>

<table>
<tr><td rowspan="4">9</td><td>谈</td><td>吐</td><td>行</td><td>止</td></tr>
<tr><td>tan</td><td>tu</td><td>xing</td><td>zhi</td></tr>
<tr><td>talk</td><td>spit</td><td>act</td><td>behave</td></tr>
<tr><td colspan="4">‘the manner of speaking and acting’</td></tr>
<tr><td rowspan="4">10</td><td colspan="2">墨</td><td colspan="2">香</td></tr>
<tr><td colspan="2">mo</td><td colspan="2">xiang</td></tr>
<tr><td colspan="2">ink</td><td colspan="2">fragrant</td></tr>
<tr><td colspan="4">‘the fragrance of ink’</td></tr>
</table>
